# COVID-19: A Rare Cause of Hemolytic Uremic Syndrome

**DOI:** 10.7759/cureus.27962

**Published:** 2022-08-13

**Authors:** Kimberly Boldig, Rishu Batra, Augusto Villegas

**Affiliations:** 1 Internal Medicine, University of Florida College of Medicine – Jacksonville, Jacksonville, USA; 2 Internal Medicine, Orange Park Medical Center, Orange Park, USA; 3 Hematology and Oncology, Florida Cancer Specialists and Research Institute, Fleming Island, USA

**Keywords:** eculuzimab in atypical hus, renal injury, covid 19, alternate pathway complement, atypical hemolytic uremia syndrome

## Abstract

Hemolytic uremic syndrome (HUS) is a multisystemic condition characterized by a triad of acute renal failure, microangiopathic hemolytic anemia, and thrombocytopenia. HUS can be classified as atypical or typical, depending upon its association with the Shiga toxin-producing Escherichia coli (STEC). Approximately 90-95% of cases are classified as typical, while only 5-10% of cases are atypical. The pathogenesis of atypical HUS (aHUS) occurs when the complement cascade is activated and causes membrane attack complex (MAC) deposition in the renal tubular epithelium. Various infectious triggers have been associated with aHUS. A new and compelling correlation to this rare and potentially deadly diagnosis of aHUS is COVID-19 infection or vaccination. We present a case of COVID-19-induced exacerbation of a patient with a known history of aHUS. In addition, we performed a literature review of previously reported COVID-19-induced aHUS cases and identified the potential pathogenesis of the disease state.

## Introduction

Hemolytic uremic syndrome (HUS) is often triggered by gastroenteritis. Infection with Escherichia coli serotypes 0157:H7 or 0104:H4 causes hemorrhagic gastroenteritis, systemic inflammation, microvascular injury, and thrombosis [1]. Rarely this systemic reaction can also occur without the bacterial infection trigger, resulting in atypical HUS (aHUS) [1]. Renal injury is an essential component of the disease state. This occurs through platelet microthrombi forming in arterioles and capillaries, causing platelet consumption and hemolytic anemia [2]. Thrombi form in glomerular vascular beds and causes endothelial cells to swell and denude. Plasma proteins, fibrin, and fibrinogen accumulate and cause further swelling, fibrinoid necrosis, and glomerular capillary aneurysms. Remodeling occurs and causes sclerosis, tubular atrophy, interstitial fibrosis, and collagen deposition [1]. The inciting factor to this cascade is still not well understood; however, the alternative complement pathway, genetic mutations, and endothelial dysfunction are believed to be important components [1]. The alternative complement pathway is not only implicated as an important component of pathogenesis but also a targetable treatment option. We present a case of COVID-19-induced aHUS exacerbation and review the literature to understand the correlation between the two disease states.

## Case presentation

We present a case of a 36-year-old woman with a previous history of aHUS, who was now in remission but undergoing treatment with eculizumab. She tested positive for COVID-19 after developing symptoms of an upper respiratory tract infection. Within three days, she presented with acute hypoxic respiratory failure and acute renal failure. Upon presentation, she was found to be hypoxic and tachycardic. She required intubation and ICU admission. Her labs were significant for hyperkalemia 5.5, acidosis with pH of 6.7, lactate 6.0, transaminitis with aspartate transaminase (AST) 270 and alanine transaminase (ALT) 182, alkaline phosphatase 219, total bilirubin 1.4, creatinine 17.9, and blood urea nitrogen (BUN) of 120 (Table 1). A CT scan of her chest demonstrated bilateral ground glass opacities, typical of a COVID-19 pneumonia infection. The rest of her infectious workup was negative.

**Table 1:** Laboratory values.

Laboratory Test	Result	Reference Range
Potassium	5.5	3.5-5.5 mEq/L
pH	6.7	7.35-7.45
Lactate	6	0.3-2.3 mEq/L
Aspartate aminotransferase (AST)	270	14-33 IU/L
Alanine aminotransferase (ALT)	182	10-42 IU/L
Alkaline phosphatase	219	32-110 U/L
Total bilirubin	1.4	0.2-1.4 mg/dL
Creatinine	17.9	0.6-1.0 mg/dL
Blood urea nitrogen	120	6-23 mg/dL
Hemoglobin	10.1	11-16 g/dL

She was started on dexamethasone for the COVID-19 infection. She also required urgent dialysis. Hematology/Oncology was consulted due to her known history of aHUS. Anemia workup was significant for hemoglobin of 10.1, low haptoglobin, and elevated lactate dehydrogenase (LDH). A peripheral smear was ordered and demonstrated schistocytes. These findings suggested a thrombotic microangiopathy and likely, an exacerbation of aHUS. This was thought to be triggered by the COVID-19 infection. The patient was then restarted on eculizumab therapy. She initially showed clinical improvement, allowing for extubation. She had improvement in BUN, creatinine, and acidosis; however, she continued to require dialysis. Her anemia worsened, requiring four units of packed RBCs during her hospital course. Worsening respiratory status resulted in reintubation and increasing respiratory support. Ultimately, she did not survive her hospital stay.

## Discussion

Complement proteins have various roles in regulating the immune system, waste disposal, angiogenesis, regenerative processes, and lipid metabolism [1]. The pathogenesis associated with aHUS results from complement dysfunction through impairment of complement regulatory proteins and/or accelerated activation of the complement pathway [3]. Complement may be activated through the classical, lectin, and alternative pathways. Specifically, the alternative complement pathway is activated by complex polysaccharides present in microorganisms. C3 convertase (C3bBb) is formed by cleaving together factors B, D, and C3. Factor P, properdin, stabilizes the C3 convertase. C3 convertase cleaves C3 while C5 convertase cleaves C5, forming C5a and C5b. Ultimately, the membrane attack complex (MAC; C5b-9, terminal complement complex) forms, facilitating target cell activation, injury, or lysis. C3a and C5a trigger proinflammatory signaling and attract neutrophils, monocytes, and macrophages, and C3b facilitates phagocytosis [1]. Soluble and membrane-bound regulators suppress complement activity. These regulators consist of factor 1 (F1), factor H (FH), and C4-binding protein (C4BP). One of the most notable regulators is FH, a cofactor to the serine protease FI, which breaks down C3b, C4b, and C3 convertase. Complement regulatory proteins are found on the surface of most human cells, allowing for accelerated complement decay and inactivation [1].

Genetic mutations in complement proteins and their regulatory proteins are found in 40-60% of patients with aHUS [1, 3]. The most common mutation affects complement factor H [3]. However, loss of function mutations in regulatory proteins and inactivating mutations in autoantibodies have all been associated with the pathogenesis of this disease. Meanwhile, a gain of function mutation may occur in complement components themselves [1]. A combination of genetic susceptibility with mutated complement proteins and exposure to an inciting event likely results in the initiation of this rare disease process by augmenting the complement pathway.

COVID-19 has been a proposed precipitator of aHUS through effects on endothelial function and direct activation of the alternative complement pathway [2, 4, 5]. COVID-19 viral RNA, nucleocapsid, and spike protein have been found to accumulate in renal tubules [2, 3]. COVID-19 spike protein activates the alternative complement pathway by competing with complement factor H [2-4, 6]. Complement cascade proteins also accumulate in the microvasculature of patients with COVID-19 [3, 7, 8]. Extensive complement activation causes MAC deposition on renal tubules. This results in microvascular endothelial cell injury, activation of the clotting pathway, fibrin deposition, and end-organ damage [3, 9, 10]. Because of complement activation, endothelial injury impairs nitric oxide's function and its regulator, vascular endothelial growth factor (VEGF). The vasodilatory, anti-inflammatory and antithrombotic effects of nitric oxide are then lost [1]. This demonstrates how COVID-19 infection may cause kidney injury by inciting inflammation, complement activation, endothelial dysfunction, and thrombotic microangiopathy [8]. 

A common presentation for aHUS includes anemia, thrombocytopenia, and renal injury. This onset may be abrupt or insidious. Laboratory testing typically shows elevated LDH, reduced haptoglobin, schistocytes on peripheral blood smear, and reduced C3 levels. Kidney injury may manifest as hypertension, proteinuria, and azotemia. Neurologic and GI symptoms may also manifest [1].

Approximately 25% of patients do not survive their acute aHUS exacerbation. Of those that survive, half develop end-stage renal disease. Patients commonly experience relapse even after renal transplants [1]. Kidney transplant itself is associated with a slight improvement in mortality and allograft failure rates ranging from 20 to 80% [1]. Other available treatment options are also limited in their efficacy. Plasma exchange or infusion improves mortality by 25% [1]. Plasma therapy works by replenishing deficient regulatory proteins or removing plasma factors that inhibit the action of regulatory proteins [1]. Pharmacologic therapy includes eculizumab, which blocks the cleavage of C5 and the formation of MAC, C5b-9. Eculizumab has shown promise in patients that have failed plasma therapy [1]. International multicenter prospective phase II trials and numerous case studies demonstrate that eculizumab treatment results in increased platelet count, improvement in kidney function, and decreased dialysis requirement [1, 11, 12].

A thorough review of the literature identified nine previous reported cases of COVID-19-associated aHUS. Seven of the cases reported were initial presentations of aHUS, and two were relapses, as seen in our case. The treatment provided to these patients and the clinical outcome are summarized in Table 2 [3, 6, 8, 13, 14]. The treatment options included eculizumab or ravulizumab, a new complement component C5 inhibitor [15].

**Table 2:** Previous described cases of aHUS and COVID-19: treatment and outcome. aHUS: Atypical hemolytic uremic syndrome.

Author	Relapse or initial diagnosis	Number of cases	Treatment	Clinical outcome
Kurian CJ et al. [3]	Relapse	1	Eculizumab	Improved
Kurian CJ et al. [3]	Initial	1	Eculizumab	Dialysis dependent
Ville S et al. [6]	Relapse	1	Eculizumab	Creatinine remained elevated
Korotchaeva J et al. [8]	Initial	1	Eculizumab	Improved
Korotchaeva J et al. [8]	Initial	1	Refused Eculizumab	Dialysis dependent
Korotchaeva J et al. [8]	Initial	1	Eculizumab	Death
Kaufeld J et al. [14]	Initial	2	Eculizumab	Improved
Gill J et al. [15]	Initial	1	Eculizumab then Ravulizumab	Improved

The COVID-19 vaccine has also been proposed as an inciting factor to complement-mediated disease presentations or exacerbations [2]. These complement-mediated conditions included paroxysmal nocturnal hemoglobinuria (PNH), autoimmune hemolytic anemia (AIHA), cold agglutinin disease (CAD), and HUS. It could be a factor contributing to HUS, although no cases have been reported. Although no cases currently describe the COVID-19 vaccine as an inciting factor of aHUS, it may be important to keep in mind for future disease presentations.

Most cases described successful treatment of COVID-19 and aHUS with continuing treatment of eculizumab. Our case provides an unfortunate instance where continued eculizumab treatment was ineffective. This may be related to the degree of respiratory compromise. Most cases previously reported in the literature described mild or no respiratory complaints but significant kidney injury. El Sissy C et al. describe five patients with COVID-19-related HUS with mild pulmonary symptoms but significant kidney injury. This presentation may indicate that complement activation occurs predominately in the kidney [5]. However, our case described a patient with significant respiratory and renal compromise. This is an essential distinction further to understand COVID-19 treatments and the effectiveness of complement inhibition. Complement activation is an important component of the pathogenesis of aHUS; however, endothelial dysfunction is also a contributing factor. It is also important to acknowledge that preventing endothelial cell dysfunction may also provide an important component of COVID-19 and aHUS treatment. Reducing endothelial cell dysfunction may occur through decreasing oxidative stress and increasing antioxidant effects. Angiotensin-converting enzyme (ACE) inhibitors, statins, and xanthine oxidase inhibitors, such as allopurinol, reduce oxidative stress [1]. Reduced oxidative stress allows increased nitric oxide and decreased endothelial cell dysfunction. Ascorbic acid, a known antioxidant, has a similar effect [1]. These may provide additional supplemental therapies to COVID-19 and aHUS treatment.

## Conclusions

This case report reviews previously published cases of COVID-19 associated with aHUS and treatment outcomes with eculizumab. Our case provides an interesting contrast as the patient ultimately died despite eculizumab treatment. The effectiveness of eculizumab treatment in aHUS may depend on the degree of respiratory compromise; as previously acknowledged by El Sissy C et al. COVID-19 is a complement-activating condition that may contribute to aHUS and kidney injury. Future studies are needed to understand how COVID-19 contributes to complement-amplifying conditions such as aHUS. Additional understanding of how these two disease states intertwine may allow for targeted treatment options within each individual diagnosis.
